# Multi-Locus Genome-Wide Association Studies of Fiber-Quality Related Traits in Chinese Early-Maturity Upland Cotton

**DOI:** 10.3389/fpls.2018.01169

**Published:** 2018-08-16

**Authors:** Junji Su, Qi Ma, Mei Li, Fushun Hao, Caixiang Wang

**Affiliations:** ^1^State Key Laboratory of Cotton Biology, Institute of Cotton Research of CAAS, Anyang, China; ^2^Cotton Research Institute, Xinjiang Academy of Agricultural and Reclamation Science, Xinjiang Academy of Agricultural and Reclamation Science, Shihezi, China; ^3^College of Plant Science and Technology, Huazhong Agricultural University, Wuhan, China; ^4^State Key Laboratory of Cotton Biology, Henan Key Laboratory of Plant Stress Biology, College of Life Science, Henan University, Kaifeng, China

**Keywords:** upland cotton, fiber quality, early maturity, multi-locus GWAS, candidate genes

## Abstract

Early-maturity varieties of upland cotton are becoming increasingly important for farmers to improve their economic benefits through double cropping practices and mechanical harvesting production in China. However, fiber qualities of early-maturing varieties are relatively poor compared with those of middle- and late- maturing ones. Therefore, it is crucial for researchers to elucidate the genetic bases controlling fiber-quality related traits in early-maturity cultivars, and to improve synergistically cotton earliness and fiber quality. Here, multi-locus genome-wide association studies (ML-GWAS) were conducted in a panel consisting of 160 early-maturing cotton accessions. Each accession was genotyped by 72,792 high-quality single nucleotide polymorphisms (SNPs) using specific-locus amplified fragment sequencing (SLAF-seq) approach, and fiber quality-related traits under four environmental conditions were measured. Applying at least three ML-GWAS methods, a total of 70 significant quantitative trait nucleotides (QTNs) were identified to be associated with five objective traits, including fiber length (FL), fiber strength (FS), fiber micronaire (FM), fiber uniformity (FU) and fiber elongation (FE). Among these QTNs, D11_21619830, A05_28352019 and D03_34920546 were found to be significantly associated with FL, FS, and FM, respectively, across at least two environments. Among 96 genes located in the three target genomic regions (A05: 27.95 28.75, D03: 34.52 35.32, and D11: 21.22 22.02 Mbp), six genes (*Gh_A05G2325, Gh_A05G2329, Gh_A05G2334, Gh_D11G1853, Gh_D11G1876*, and *Gh_D11G1879*) were detected to be highly expressed in fibers relative to other eight tissues by transcriptome sequencing method in 12 cotton tissues. Together, multiple favorable QTN alleles and six candidate key genes were characterized to regulate fiber development in early-maturity cotton. This will lay a solid foundation for breeding novel cotton varieties with earliness and excellent fiber-quality in the future.

## Introduction

Upland cotton (*Gossypium hirsutum* L.), a tetraploid plant, is the most important natural-fiber crop. It is widely cultivated in the world and supplies more than 95% of the global fiber yield due to its extensive adaptive ability and high productivity (Chen et al., 2007). Upland cotton cultivars can be divided into early-, middle- and late- maturity varieties, according to the duration of growth period. Early-maturity (short-season) cotton is an ecological type with a relatively short growing period (Yu et al., 2005; Song et al., 2015). It is suited for wheat-cotton, barley-cotton and rape-cotton double cropping patterns in cotton growing areas of Yellow River Region (YRR) and Yangzi River Region (YZRR), and is also fit for single cropping production in the early-maturity areas of Northwest Inland Region (NIR) and the Northern Specific Early-Maturity Region (NSEMR), with the short frost-free period in China (Yu et al., 2005; Song et al., 2015). Additionally, mechanized harvesting of cotton after good ripening is very common in NIR. The cotton varieties appropriate for mechanical harvesting should have earlier maturing characteristics, especially for early and concentrated boll-opening traits, compared with those suitable for manual harvesting (Bao et al., 2014; Feng et al., 2017). Therefore, the early-maturity upland cotton varieties are becoming more and more important in Chinese cotton production.

Currently, farmers gained increased economic benefits after using new production patterns of double cropping and mechanical harvesting; whereas application of these cultivation measures need early-maturity cotton (Du et al., 2015; Dai et al., 2017; Lu et al., 2017). Owing to their great necessity, a series of early-maturity cotton varieties, such as “Liaomian,” “Zhongmiansuo,” and “Xinluzao,” were developed and released in recent 40 years in China. However, their fiber qualities were relatively poor compared with those of middle- and late- maturity cotton varieties. Therefore, it is crucial to improve fiber quality of early-maturity cotton varieties.

To meet human higher needs for improving textile products, it is also essential for researchers to focus on fiber-quality improvement of early-maturity cotton in future. However, it is difficult to improve fiber quality of early-maturity cotton by means of traditional breeding strategy because of the significant negative correlation between earliness and excellent-quality fiber (Song et al., 2005; Fan et al., 2006). The rapid development of genotyping techniques based on simple sequence repeat (SSR) and single nucleotide polymorphism (SNP) markers provided an alternative method to improve the efficiency of crop breeding. Generally, marker-assisted selection (MAS) is a high-efficiency and economical approach for modern breeding, compared with the traditional phenotyping breeding (Lande and Thompson, 1990). Researchers have spent a great amount of time and effort on mapping quantitative trait loci (QTL) by using linkage analysis. Over the last two decades, a number of cotton earliness-related QTL have been identified via linkage mapping (Fan et al., 2006; Li et al., 2013; Jia et al., 2016). Compared to the studies evaluating cotton early maturity, far too many investigations have been conducted to identify genetic signatures for fiber quality. A recent meta-QTL analysis suggested that approximately one thousand QTL for fiber-related traits have been detected in intraspecific upland cotton populations (Said et al., 2015), and a few near-term studies have added new QTL for cotton fiber quality (Shang et al., 2015; Tan et al., 2015; Tang et al., 2015; Fang X. et al., 2017).

A genome-wide association study (GWAS) is a wonderful supplement to QTL mapping, and it has been widely used in upland cotton in recent years (Su et al., 2016a, 2018; Fang L. et al., 2017; Huang et al., 2017; Sun et al., 2017; Ma Z. et al., 2018). Although there are a lot of reports on GWAS for cotton earliness and fiber-quality related traits in the past ten years (Zeng et al., 2009; Zhang et al., 2013; Cai et al., 2014; Nie et al., 2016; Su et al., 2016a,b; Sun et al., 2017; Ma Z. et al., 2018), few GWAS investigations have been conducted on fiber-quality related traits in early-maturity upland cotton. In the previous studies, the majority of QTL or quantitative trait nucleotides (QTNs) for fiber quality are mainly derived from germplasms of *G. barbadense* and late-maturity *G. hirsutum*, they are not convenient for use in fiber-quality improvement of early-maturity cotton. Therefore, it is needed to identify QTNs and candidate genes associated with fiber quality in the panel consisting of early-maturity upland cotton accessions.

To date, a lot of single-locus GWAS (SL-GWAS) have been reported in upland cotton (Zeng et al., 2009; Zhang et al., 2013; Nie et al., 2016; Su et al., 2016a,b,c; Sun et al., 2017; Ma Z. et al., 2018). The SL-GWAS methods are involved in multiple testing, and Bonferroni correction is frequently adopted to control the false positive rate. However, this correction is very stringent, thus some important loci cannot be detected, especially for large error in the phenotypic measurement in field experiments (Tamba et al., 2017). To overcome this issue, multi-locus GWAS (ML-GWAS) methodologies have been developed. They include mrMLM (Wang et al., 2016), FASTmrMLM (Tamba and Zhang, 2018), ISIS EM-BLASSO (Tamba et al., 2017), FASTmrEMMA (Wen et al., 2017), pLARmEB (Zhang et al., 2017), and pKWmEB (Ren et al., 2018). Additionally, to decrease the false positive rate, a combination of several ML-GWAS methods have been applied in previous studies (Wu et al., 2016; Misra et al., 2017; Ma L. et al., 2018).

In this study, ML-GWAS for fiber-quality related traits were conducted in a panel composed of 160 early-maturing cotton accessions. The main objective of our study was to discover the favorable QTN allelic variations and some potential candidate genes controlling fiber quality in the early-maturity upland cotton. This investigation will lay a foundation for breeding new cotton varieties with earliness and excellent fiber quality in the future.

## Materials and methods

### Plant materials

A natural population consisting of 160 Chinese early-maturity upland cotton accessions were generated (Table S1). These accessions were sampled from the germplasm gene bank of the Cotton Research Institute of the Chinese Academy of Agricultural Sciences (CRI-CAAS). The germplasms fell into three groups based on cotton-planting regions in China. Specifically, 81, 58, and 21 accessions were from the YRR, NIR and NSEMR, respectively. All the accessions have relatively short whole growing period (ranging from 100 to 120 days).

### Field experiments

A collection of 160 early-maturity upland cotton accessions was evaluated under four environmental conditions (2 locations × 2 years): Anyang, Henan, China (36.13°N, 114.80°E) in 2014 and 2015 (designated AY-2014 and AY-2015, respectively), and Shihezi (SHZ), Xinjiang, China (44.52°N, 86.02°E) in 2014 and 2015 (designated SHZ-2014 and SHZ-2015, respectively). The field experiments were arranged in a randomized complete block design with three replications. At AY, each accession was sown in a single-row plot with about 20 plants, while at SHZ, each accession was planted in double-row plots with about 30 plants. The field trials at SHZ were performed with drip irrigation under plastic film conditions, whereas the plots at AY were furrow irrigated as needed. The experimental field management measures were full accordance with local agronomic practices.

### Phenotyping and data analysis

After mature, a total of 20 naturally opened bolls, as a cotton fiber sample, were handly picked from central part of the plants from each accession in each replicate every year. Fiber samples weighing 10~15 g lint cotton were then measured for fiber property determination using an HVI-MF 100 instrument (User Technologies, Inc., USTER, Switzerland) at the Cotton Fiber Quality Inspection and Testing Center of the Ministry of Agriculture, Anyang, China. The following fiber-quality related traits were evaluated: 50% fiber span length (FL, mm), fiber strength (FS, cN.tex^−1^), fiber micronaire (FM), fiber uniformity (FU, %) and fiber elongation (FE, %). The analysis of variance (ANOVA) for phenotypic data was conducted using the SPSS22.0 software.

### SNP genotyping

Genomic DNA was isolated from young leaf tissue of all accessions using a modified cetyltrimethylammonium bromide (CTAB) method as described by Paterson et al. (1993). Reduced-representation DNA sequences of 160 early-maturity cotton accessions have been obtained by specific-locus amplified fragment sequencing (SLAF-seq) approach with coverage of approximate 5.50×. To mine the SNPs with higher quality, the raw reads were mapped to the *G. hirsutum* L. TM-1 genome v 1.1 (Zhang et al., 2015) using BWA software (Li and Durbin, 2009). The GATK (McKenna et al., 2010), and SAMTools (Li et al., 2009) packages were used for SNP calling. The filtered SNPs, with a missing rate <10% and a minor allele frequency (MAF) ≥ 0.05, were reserved and used for the subsequent analysis.

### Clustering analysis, population structure and linkage disequilibrium (LD) analysis

A neighbor-joining phylogenetic tree among 160 individuals was constructed using the filtered SNPs by the Tassel 5.2 software (Bradbury et al., 2007). The population structure was analyzed using a principal component analysis (PCA) approach with the Tassel 5.2 program (Bradbury et al., 2007). LDs between SNPs were estimated as the squared correlation coefficient (R^2^) of alleles using the Tassel 5.2 tool (Bradbury et al., 2007). The *R*^2^-values were calculated within a 0- to 10-cM window.

### Genome-wide association study and allelic variation analysis

Six ML-GWAS methods, including the mrMLM, FASTmrMLM, FASTmrEMMA, pLARmEB, pKWmEB, and ISIS EM-BLASSO, were used in this study. The mrMLM is a multi-locus model including markers selected from the rMLM method with a less stringent selection criterion (Wang et al., 2016). The FASTmrMLM reduces the running time in mrMLM by more than 50%, and also shows slightly high statistical power in QTN detection, high accuracy in QTN effect estimation and low false positive rate as compared to mrMLM (Tamba and Zhang, 2018). FASTmrEMMA is a fast multi-locus random-SNP-effect EMMA model, which is more powerful in QTN detection and model fit (Wen et al., 2017). The pLARmEB integrates least angle regression with empirical Bayes to perform ML-GWAS under polygenic background control (Zhang et al., 2017). The pKWmEB retains the high power of Kruskal–Wallis test, and provides QTN effect estimates and effectively controls false positive rate (Ren et al., 2018). The ISIS EM-BLASSO has the highest empirical power in QTN detection and the highest accuracy in QTN effect estimation, and it is the fastest, as compared with EMMA and mrMLM (Tamba et al., 2017). All parameters were set at default values, and the critical thresholds of significant association for all the above six methods were set at LOD = 3.00 (Wang et al., 2016; Tamba et al., 2017; Wen et al., 2017; Zhang et al., 2017; Ren et al., 2018; Tamba and Zhang, 2018).

The phenotypic-effect value of each allelic variation was calculated by the phenotypic data over the accessions with each type, and box plots of the relative phenotypic data were produced using the R software.

### Prediction of potential candidate genes

Putative candidate genes were identified by physical positions of significant trait-associated SNP loci in the *G. hirsutum* L. reference genomes v1.1 (Zhang et al., 2015). According to LD decay distance, the interval for the prediction of candidate genes for the significant SNP loci was determined. The genes distributed in these regions were collected. Transcriptome sequencing data from 12 upland cotton tissues (including fiber in 5, 10, 20, 25 DPA (days post anthesis), root, stem, leaf, torus, calycle, cotyledon, petal and pistil) were available on the cotton website (https://cottonfgd.org/). Heat maps of the putative candidate gene expression patterns were drawn using the R package “pheatmap.” The biological functions of putative candidate genes were annotated by gene ontology (GO) items on the cotton website (https://cottonfgd.org/).

## Results

### SNP genotyping

To gain insight into the genetic bases of fiber-quality related traits, 160 early-maturity upland cotton accessions were performed using SLAF-seq, and a complete set of markers containing 72,792 high-quality SNPs was explored by filtering according to the stringent quality control. These detected markers consisted of 47,594 and 25,198 SNPs in the At and Dt chromosomes respectively, and were unevenly distributed on all the 26 chromosomes of upland cotton. Moreover, the SNP loci with maximal number were identified on chromosome A10 (5013), while those with the minimal number were detected on chromosome D04 (1479). The average marker density was about one SNP per 28.10 kb genomic regions. The greatest marker density was found on chromosome A10 with one SNP per 20.12 kb, while the smallest marker density was seen in chromosome D05, with one SNP per 39.93 kb (Table 1).

**Table 1:** Statistics of SNPs.

**Chr**.	**Number of SNPs**	**Density of SNP (kb/SNP)**	**Chr**.	**Number of SNPs**	**Density of SNP (kb/SNP)**
A01	4410	22.65	D01	1873	32.81
A02	3460	24.12	D02	1846	36.45
A03	3367	29.78	D03	1655	28.21
A04	2485	25.32	D04	1479	34.79
A05	3320	27.73	D05	1551	39.93
A06	4414	23.37	D06	1991	32.29
A07	3388	23.10	D07	1658	33.36
A08	4703	22.03	D08	2699	24.41
A09	2443	30.70	D09	1635	31.19
A10	5013	20.12	D10	2240	28.29
A11	3817	24.45	D11	2466	26.80
A12	2996	29.20	D12	2016	29.32
A13	3778	21.16	D13	2089	28.98

*Chr., chromosome, according to the upland cotton reference genome (Zhang et al., 2015)*.

### Phenotypic variation

**Figure 1:**
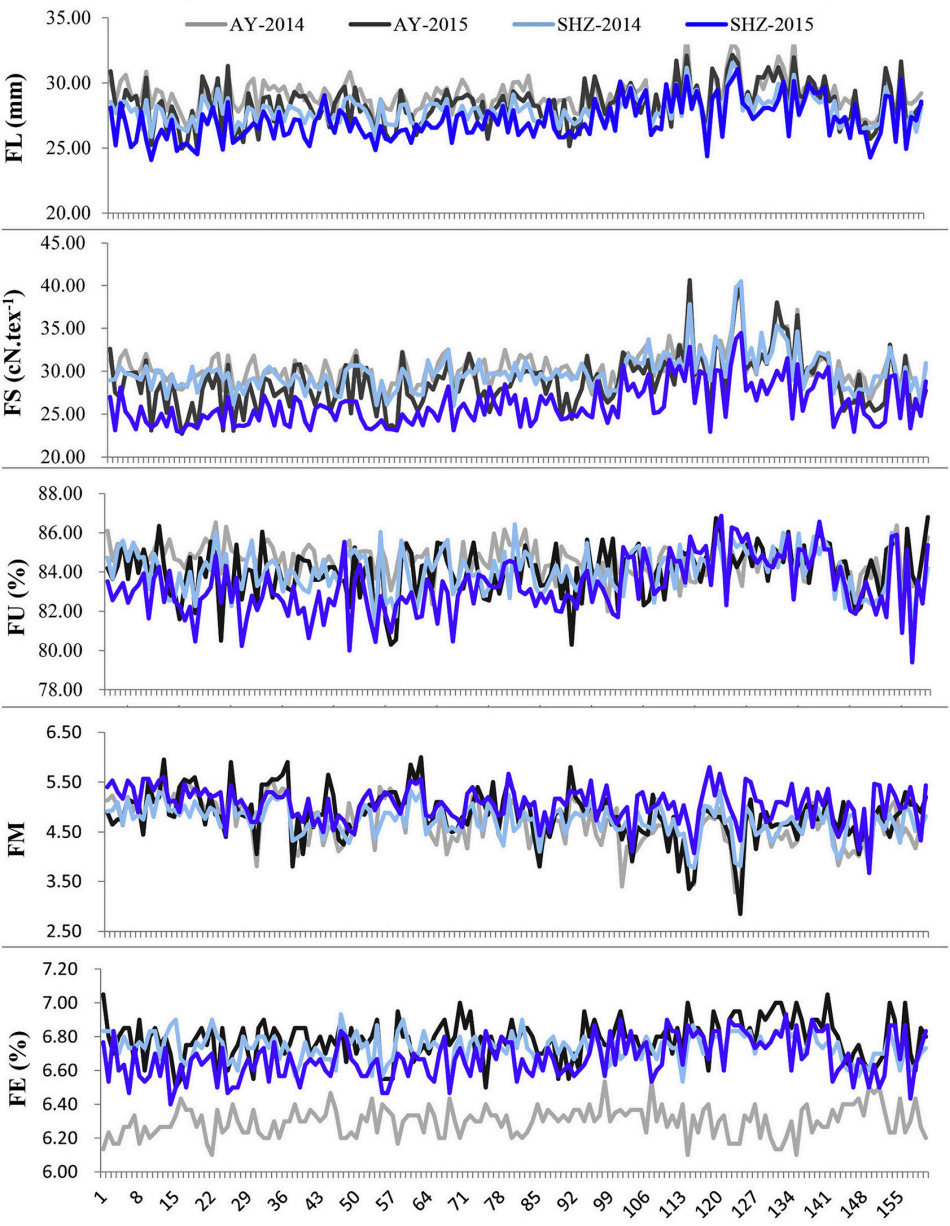
Phenotypic distributions of five fiber-quality related traits of 160 early-maturity upland cotton accessions in four cultivating environments.

To examine whether significant phenotypic variances exist in the fibers among the 160 upland cotton accessions, the five fiber-quality related traits including FL, FS, FU, FM and FE were examined. The results showed that the parameters of fibers from different accessions were quite diverse (Table 2). For instance, the FL ranged from 24.07 to 33.69 mm, with a mean of 28.09 mm, the FS had a great variation ranging from 22.70 to 40.65 cN.tex^−1^, and the FM from four environments varied from 2.50 to 6.00, with an average value of 4.83. Additionally, the FU and FE had wide distributions and variations (Table 2, Figure 1). These results indicate that early-maturity cotton varieties had broad variation in fiber-quality related traits under different planting conditions.

**Table 2:** Phenotypic distribution range of five fiber-quality related traits of 160 early-maturity upland cotton accessions.

**Traits**	**Environments**	**Mean**	**Min**	**Max**	***SD***	**CV (%)**
FL (mm)	AY-2014	29.08	26.31	33.69	1.25	4.31
	AY-2015	28.33	24.80	32.15	1.65	5.82
	SHZ-2014	27.86	25.79	31.53	1.08	3.88
	SHZ-2015	27.10	24.07	31.07	1.47	5.43
FS (cN.tex^−1^)	AY-2014	30.40	26.07	39.43	2.22	7.31
	AY-2015	28.75	22.70	40.65	3.15	10.97
	SHZ-2014	29.85	25.94	40.54	2.24	7.51
	SHZ-2015	26.07	22.87	34.47	2.44	9.35
FU (%)	AY-2014	84.60	82.00	86.53	0.86	1.02
	AY-2015	84.06	80.30	86.80	1.29	1.54
	SHZ-2014	84.04	80.80	86.43	1.11	1.32
	SHZ-2015	83.29	79.40	86.87	1.44	1.72
FM	AY-2014	4.68	3.28	5.78	0.50	10.61
	AY-2015	4.85	2.85	6.00	0.50	10.25
	SHZ-2014	4.73	3.78	5.39	0.32	6.68
	SHZ-2015	5.05	3.67	5.80	0.35	6.98
FE (%)	AY-2014	6.30	6.10	6.53	0.09	1.39
	AY-2015	6.78	6.50	7.05	0.12	1.77
	SHZ-2014	6.74	6.50	6.93	0.08	1.22
	SHZ-2015	6.68	6.40	6.93	0.12	1.79

*FL, fiber length; FS, fiber strength; FM, fiber micronaire; FU, fiber uniformity; FE, fiber elongation; Max, maximum; Min, minimum; SD, standard deviation; CV, coefficient of variation; AY, Anyang; SHZ, Shihezi*.

We observed that the phenotypic values of FL and FS at Anyang (AY) were significantly lower than those at Shihezi (SHZ). By contrast, there were no significant differences in FM and FU between the two locations. The FE values in AY-2014 were also strikingly lower than those in other environments (Table 2, Figure 1). Furthermore, the statistically significant differences (*P* < 0.001) were observed among genotypes, environments, and the genotype × environment interactions on all the five target traits (Table S2). These data suggest that the five fiber-quality related traits were significantly influenced by the environmental conditions.

### Population structure and linkage disequilibrium analysis

**Figure 2:**
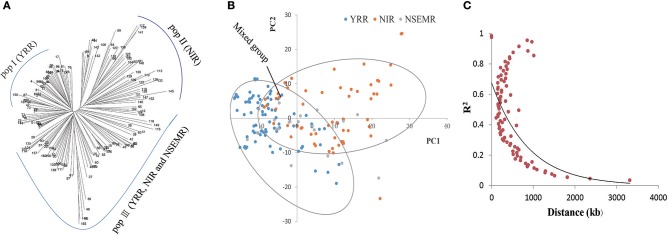
Phylogenetic tree, population structure and LD decay of 160 early-maturity upland cotton accessions. **(A)** Neighbor-joining phylogenetic tree of all cotton accessions. The pop III was a mixed group including YRR, NIR and NSEMR. **(B)** Principal component analysis (PCA) of the association panel. **(C)** The entire genome LD decay of the population.

To understand the phylogenetic relationship of the 160 upland cotton genotypes, a neighbor-joining phylogenetic tree was conducted based on their genetic distances, which derived from the SNP differences in these accessions. The population could be divided into three different groups, designated pop I (YRR, with 54 accessions), pop II (NIR, with 44 accessions) and pop III (YRR, NIR and NSEMR, with 62 accessions), respectively (Figure 2A). Furthermore, we found that there are an intimate genetic relationship between NSEMR accessions and the early varieties from YRR and NIR, which were mainly assigned to pop III, while the recent accessions from YRR and NIR belonged to pop I and pop II, respectively. These findings imply that early-maturity accessions in YRR and NIR might derive from NSEMR varieties in upland cotton.

Next, the population structure of the panel was analyzed using a PCA on the basis of the identified SNPs. Three conceivable subpopulations were separated by PC1 and PC2 (Figure 2B). Similarly, YRR, NIR and mixed group (YRR, NIR and NSEMR) were respectively distinguished via PCA. Based on the results from both the phylogenetic tree and PCA, the panel was separated into three groups (Figures 2A,B).

To examine the LD decay distance in the panel, its decay rate was estimated using the SNPs. The result showed that the genome-wide LD decay rate of the natural population was approximately 400 kb, where the R^2^ drops to half of the maximum value (Figure 2C). Due to the average marker density with one SNP per 28.10 kb (Table 1), we concluded that these markers were sufficiently dense for detecting the associated QTNs.

### Multi-locus genome-wide association studies

**Figure 3:**
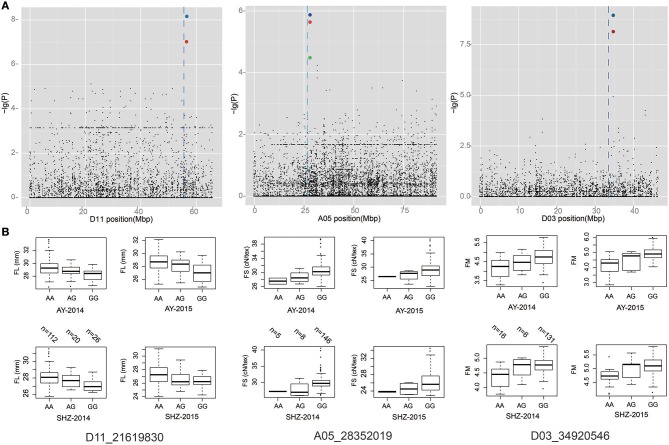
Local Manhattan plot (top), and box plots for the fiber-quality related traits (bottom). **(A)** Manhattan plots of FL, FS, and FM on chromosome A05, D03, and D11, respectively. **(B)** Box plots of the significant QTNs (D11_21619830, A05_28352019, and D03_34920546). Each dot represents an SNP. The vertical dashed lines indicate the genomic region containing the significant QTNs. The red and blue circles mark the significant QTNs.

A total of 70 significant QTNs were simultaneously detected to be associated with the above five objective traits by at least three multi-locus GWAS (ML-GWAS) methods (Table 3). Among these QTNs, 16, 20, 9, 16, and 9 were found to be associated with FL, FS, FM, FU, and FE, respectively. Among the 70 significant QTNs, three were simultaneously presented across at least two environments (Table 3). One (D11_21619830) for FL, with a high proportion of total phenotypic variance explained by the QTN (2.35~11.07%), was found simultaneously in the two planting environments (AY-2014 and SHZ-2014) (Figure 3A, Table 3). Note that this QTN was detected by three methods (mrMLM, ISIS EM-BLASSO and pLARmEB) in AY-2014, and by four methods (mrMLM, FASTmrMLM, pLARmEB and ISIS EM-BLASSO) in SHZ-2014. Another QTN (A05_28352019) for FS was found by four methods (mrMLM, FASTmrMLM, pLARmEB and pKWmEB) in AY-2014 and by three methods (mrMLM, FASTmrMLM and pLARmEB) in SHZ-2014 (Figure 3A, Table 3). Most meaningfully, the QTN (D03_34920546) for FM was presented simultaneously in three environments (AY-2014, AY-2015 and SHZ-2015), and was detected at AY (2014 and 2015) by all the six ML-GWAS methods (Figure 3A, Table 3). In conclusion, the three identified QTNs (A05_28352019, D03_34920546 and D11_21619830), might be some steady major QTNs controlling the target traits.

**Table 3:** The significant QTNs for five fiber-quality related traits detected simultaneously by using three or more multi-locus GWAS methods.

**Traits**	**QTN ID**	**Env**.	**Pos.(Mb)**	**Chr**.	**LOD**	***R*^2^ (%)**	**ML-GWAS Methods**
FL	A02_8008893	AY-2014	8.01	A02	3.69–6.42	5.66–15.50	2, 3, 5
	A07_14808135	AY-2014	14.80	A07	3.10–5.22	6.97–12.22	1, 2, 5, 6
	A09_5999806	AY-2014	6.00	A09	3.23–7.46	2.47–12.33	1, 2, 4
	A11_77306905	AY-2014	77.31	A11	3.90–6.75	2.11–9.81	1, 2, 4, 5
	D01_38549510	AY-2014	38.55	D01	4.32–4.90	2.83–9.30	1, 2, 5
	D03_37661749	AY-2014	37.66	D03	3.56–5.11	1.71–6.70	1, 2, 4, 5, 6
	D08_44134483	AY-2014	44.13	D08	4.30–4.48	1.98–6.02	1, 2, 5
	D11_21619830^*^	AY-2014	21.62	D11	4.15–5.29	2.35–9.18	1, 3, 5
	D03_41720764	AY-2015	41.72	D03	3.00–5.09	7.08–13.15	1, 2, 4, 6
	A07_71351661	SHZ-2014	71.35	A07	4.95–5.94	2.84–11.07	1, 4, 5
	D11_21619830^*^	SHZ-2014	21.62	D11	3.99–5.45	2.79–11.32	1, 2, 3, 5
	A01_20468506	SHZ-2015	20.47	A01	3.88–4.72	8.71–9.84	1, 3, 5
	A07_5555999	SHZ-2015	5.56	A07	3.06–4.90	5.24–13.44	1, 2, 5, 6
	A09_56201893	SHZ-2015	56.20	A09	3.07–5.07	2.22–6.81	2, 3, 4, 5, 6
	D06_15590320	SHZ-2015	15.59	D06	3.23–5.64	2.31–6.62	2, 3, 4, 5, 6
	D13_20291732	SHZ-2015	20.29	D13	3.01–3.49	10.41–17.71	2, 5, 6
FS	A05_28352019^*^	AY-2014	28.35	A05	4.21–6.09	4.73–14.91	1, 2, 5, 6
	A01_3833158	AY-2015	3.83	A01	4.70–7.47	8.01–8.89	1, 5, 6
	A11_48101548	AY-2015	48.10	A11	3.60–4.21	7.86–8.44	1, 2, 6
	D01_53611999	AY-2015	53.61	D01	4.13–4.95	4.73–7.43	2, 5, 6
	D08_54727428	AY-2015	54.73	D08	3.58–10.36	2.23–9.81	1, 2, 6
	D08_63040058	AY-2015	63.04	D08	3.97–4.91	3.70–5.98	4, 5, 6
	A05_28352019^*^	SHZ-2014	28.35	A05	3.14–6.70	4.03–8.57	1, 2, 5
	A05_81758788	SHZ-2014	81.76	A05	3.37–6.73	2.59–6.47	1, 2, 5, 6
	A07_71351661	SHZ-2014	71.35	A07	3.14–7.25	3.36–6.33	1, 2, 4, 5
	A08_71454278	SHZ-2014	71.45	A08	3.27–5.88	6.75–11.73	2, 5, 6
	A09_30635120	SHZ-2014	30.64	A09	3.28–5.78	5.77–11.18	1, 2, 5
	A11_85908613	SHZ-2014	85.91	A11	4.32–9.06	15.66–18.87	2, 3, 5
	D01_17607059	SHZ-2014	17.61	D01	3.55–13.41	3.98–13.41	2, 5, 6
	D06_15477129	SHZ-2014	15.48	D06	5.27–6.94	5.60–7.21	1, 2, 5
	D07_45641817	SHZ-2014	45.64	D07	3.36–5.33	0.93–1.33	1, 2, 5
	A09_56201893	SHZ-2015	56.20	A09	3.14–4.33	1.80–4.39	3, 4, 5
	A13_60864258	SHZ-2015	60.86	A13	3.77–5.28	3.63–8.03	1, 2, 4
	D01_53662824	SHZ-2015	53.66	D01	4.35–5.55	2.69–7.99	3, 5, 6
	D06_15590320	SHZ-2015	15.59	D06	3.13–4.24	0.88–4.39	1, 2, 4, 5, 6
	D12_51137790	SHZ-2015	51.14	D12	3.14–5.64	4.51–14.69	3, 5, 6
FM	A03_97922050	AY-2014	97.92	A03	4.01–7.02	5.00–6.91	1, 2, 5, 6
	A05_31842417	AY-2014	31.84	A05	3.04–5.46	3.53–15.10	1, 2, 3, 4, 5, 6
	D03_34920546^*^	AY-2014	33.49	D03	4.18–9.66	5.58–13.87	1, 2, 3, 4, 5, 6
	D03_34920546^*^	AY-2015	34.92	D03	9.31–12.88	16.69–29.90	1, 2, 3, 4, 5, 6
	A06_5267401	SHZ-2014	35.27	A06	3.54–5.66	3.79–8.48	1, 3, 4, 6
	D03_34920546^*^	SHZ-2014	34.92	D03	3.86–5.26	5.89–17.95	1, 3, 5, 6
	D09_23399960	SHZ-2014	23.40	D09	3.99–5.78	5.12–11.08	1, 2, 3
	D11_1353254	SHZ-2014	1.35	D11	3.01–6.58	3.35–13.14	1, 2, 3, 4, 5
	D04_42032569	SHZ-2015	42.03	D04	3.59–4.24	4.81–11.96	2, 3, 5
FU	D07_43127704	AY-2014	43.13	D07	4.61–6.30	4.40–14.6	1, 2, 3, 4, 5
	A01_84353970	AY-2015	84.35	A01	3.12–3.77	4.24–4.68	1, 3, 5
	A05_65094242	AY-2015	65.09	A05	3.13–4.43	2.58–12.3	2, 3, 4
	A05_81758788	AY-2015	81.76	A05	3.82–8.50	5.32–9.88	1, 2, 5
	A09_577845	AY-2015	0.58	A09	3.57–5.04	2.34–4.94	1, 2, 3
	A11_90350675	AY-2015	90.35	A11	4.13–5.03	3.10–3.89	1, 2, 3
	D11_22135870	AY-2015	22.14	D11	3.00–6.25	4.17–6.68	1, 2, 5
	D12_29952510	AY-2015	29.95	D12	3.77–4.33	5.70–9.85	3, 4, 5
	A06_56710162	SHZ-2014	56.71	A06	3.51–5.14	4.32–12.49	1, 2, 3, 4, 5
	A07_71351661	SHZ-2014	71.35	A07	3.32–4.12	4.68–7.58	1, 2, 3
	A10_91235190	SHZ-2014	91.24	A10	4.00–8.37	6.79–12.89	1, 2, 3, 4, 5
	D08_44134483	SHZ-2014	44.13	D08	3.00–6.49	4.30–9.44	1, 2, 3, 4, 5
	D11_22044769	SHZ-2014	22.04	D11	3.34–7.46	9.50–23.35	1, 2, 4, 5, 6
	A04_57215161	SHZ-2015	57.22	A04	3.54–4.07	11.12–15.91	1, 2, 6
	D03_41720764	SHZ-2015	41.72	D03	4.05–6.07	6.67–9.88	1, 2, 3, 6
	D10_56039747	SHZ-2015	56.04	D10	4.41–7.04	7.97–12.76	2, 3, 4, 5, 6
FE	A11_77306905	AY-2014	77.31	A11	3.15–7.78	4.58–12.40	1, 2, 3, 4, 5, 6
	D07_21396263	AY-2014	21.40	D07	6.24–7.56	5.81–10.32	1, 2, 5
	D07_43127704	AY-2014	43.13	D07	4.15–6.40	1.47–6.99	1, 3, 5, 6
	A01_60422471	AY-2015	60.42	A01	3.67–5.88	3.65–17.78	1, 2, 4
	A05_22406870	AY-2015	22.41	A05	3.148–3.80	0.83–8.12	1, 2, 5
	A09_46286411	AY-2015	46.29	A09	3.84–6.68	7.93–10.45	1, 2, 4
	D01_53662824	AY-2015	53.66	D01	3.28–6.24	7.09–14.52	1, 3, 4, 6
	D04_32424503	AY-2015	32.42	D04	3.02–8.53	3.35–5.79	1, 2, 3, 5
	A09_56201893	SHZ-2015	56.20	A09	3.40–4.12	4.75–12.57	3, 4, 6

*FL, fiber length; FS, fiber strength; FM, fiber micronaire; FU, fiber uniformity; and FE, fiber elongation; QTN, quantitative trait nucleotide; Env., Environment; Chr., chromosome; Pos*.,

*position; AY, Anyang; SHZ, Shihezi. LOD value indicates the significance levels and R^2^ (%) indicates the percentage of phenotypic variation explained by each QTN*.

*mrMLM, FASTmrMLM, ISIS EM-BLASSO, FASTmrEMMA, pLARmEB and pKWmEB are marked by 1 to 6, respectively*.

* Indicates the significant QTNs presented simultaneously across at least two environments

### Identification of favorable allelic variations

To identify favorable alleles of QTNs for target traits, we focused on the above 3 steady QTNs, which exhibited the maximum LOD, –lg(*P*) value and phenotypic variation. The striking QTN D11_21619830 presented three types of allele (AA, AG and GG), and the accessions with the favorable allele AA (*n* = 112) showed significantly higher FL than those with the GG (*n* = 26) and AA (*n* = 20) alleles (Figure 3B). Moreover, we found that QTN A05_28352019 had three types of allelic variation AA, AG and GG, respectively, where the average FS of the favorable allele GG (28.89 cN.tex^−1^) was higher than those of the AA (26.26 cN.tex^−1^) and AG (26.98 cN.tex^−1^) (Figure 3B). Additionally, the peak QTN (D03_34920546) had three allelic variations (AA, AG and GG), and the accessions with the GG variation showed higher FM than those with the alternate AA variation. Considering the most excellent level of FM (3.70~4.20) for spinning, allele AA of the peak QTN could be regarded as the favorable allele with the mean FM value of 4.28, whereas the corresponding type GG was the unfavorable allele with the mean FM value of 4.89 (Figure 3B). These findings indicated that the fibers of the accessions with favorable allelic variations were clearly improved compared to those of the accessions with unfavorable allelic variations.

### Prediction of candidate genes

**Figure 4:**
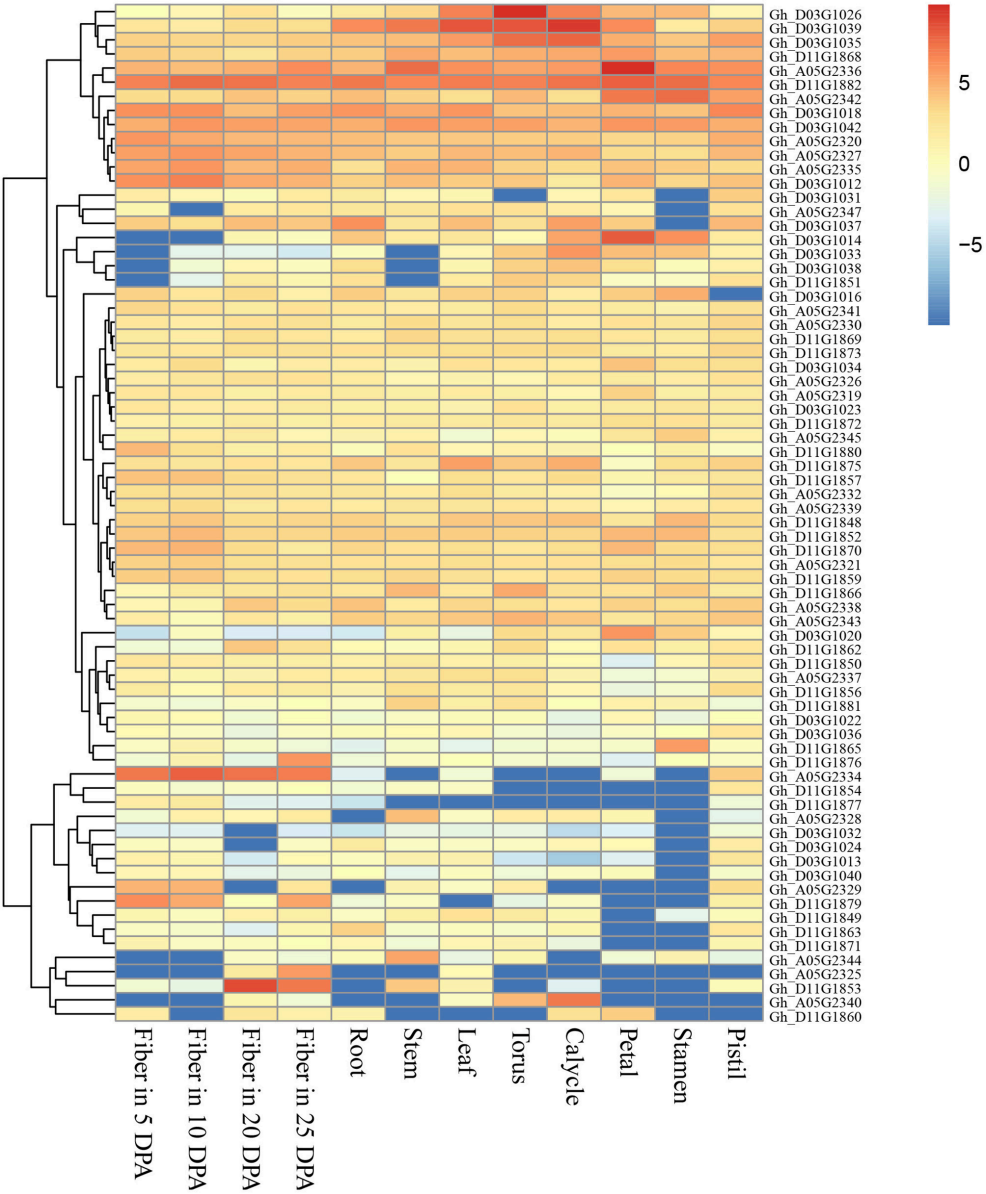
Heat map of expression level of the 72 genes in 12 upland cotton tissues. The red indicates high expression, and the blue shows low expression.

The genomic regions (±400 kb around the associated QTNs) of QTN-linked candidate genes were adopted according to the genome-wide LD decay distances (about 400 kb) in this study. Thus, three target regions of the candidate genes were determined as A05: 27.95–28.75, D03: 34.52–35.32, and D11: 21.22–22.02 Mbp, and a total of 29, 32 and 35 genes were presented respectively in the above regions, according to upland cotton reference genome v1.1 (Zhang et al., 2015; Table S3). Furthermore, we observed that the expression of 72 genes of them was clearly increased in 12 cotton tissues using RNA-Seq (Figure 4). Among these genes, *Gh_A05G2325, Gh_A05G2329, Gh_A05G2334, Gh_D11G1853, Gh_D11G1876*, and *Gh_D11G1879*, were highly expressed in the fiber. Notably, *Gh_A05G2334* was dominantly expressed in all the four fiber samples; *Gh_D11G1853* was mainly expressed in fibers of 20 and 25 DPA; and *Gh_D11G1876* and *Gh_A05G2325* was preferentially expressed in fiber of 25 DPA; whereas *Gh_A05G2329* and *Gh_D11G1879* had the maximum expression level in the fibers of 5 and 10 DPA, respectively. Also, the transcriptional abundances of *Gh_D03G1012* and *Gh_A05G2335* were slightly higher in fibers than in the other tissues (Figure 4). These results suggest that the six genes (*Gh_A05G2325, Gh_A05G2329, Gh_A05G2334, Gh_D11G1853, Gh_D11G1876*, and *Gh_D11G1879*) might play important roles in controlling fiber quality of early-maturity upland cotton.

To further understand thoroughly the above six putative candidate genes for target traits, their biological functions were annotated by gene ontology (GO) items. Three genes (*Gh_A05G2334, Gh_D11G1876*, and *Gh_D11G1879*) were annotated as transcription factors, such as sequence-specific DNA binding, DNA-binding transcription factor activity and regulation of transcription (Table 4). *Gh_A05G2334* encoded the agamous-like MADS-box protein AGL11 which likely plays roles in many aspects of plant growth and development (Rounsley et al., 1995). These results indicate that the putative candidate genes may regulate fiber development by DNA-binding transcription factors in early-maturity upland cotton.

**Table 4:** The biological function annotations of the six putative candidate genes for five fiber-quality related traits.

**Gene ID**	**Name**	**Chr**.	**Description**	**Function annotations**
*Gh_A05G2325*		A05	Non-specific lipid-transfer protein 2	
*Gh_A05G2329*		A05		
*Gh_A05G2334*	*AGL11*	A05	Agamous-like MADS-box protein AGL11	DNA binding; DNA-binding transcription factor activity; regulation of transcription; protein dimerization activity
*Gh_D11G1853*	*ephx3*	D11	Epoxide hydrolase 3	
*Gh_D11G1876*	*GBF1*	D11	G-box-binding factor 1	DNA-binding transcription factor activity; regulation of transcription; sequence-specific DNA binding; nutrient reservoir activity
*Gh_D11G1879*	*ATHB-40*	D11	Homeobox-leucine zipper protein ATHB-40	DNA binding; DNA-binding transcription factor activity; regulation of transcription; transcription regulatory region sequence-specific DNA binding

*Function annotations of putative candidate genes were conducted by gene ontology (GO) items on the cotton website (https://cottonfgd.org/); Chr.: Chromosome*.

## Discussion

### The origin and domestication of chinese early-maturity upland cotton

To exploit the limited natural resources and increase economic income of cotton producers, it is especially necessary to make use of the double cropping systems and mechanical harvesting in the major cotton growing regions in China. Thus, early-maturity cotton cultivars are needed. Indeed, early-maturity cotton varieties are attracting much attention from many cotton growers and breeders. Fiber characters are complicated and comprehensive traits regulated by a lot of QTL and influenced easily by many external factors (Ulloa and Meredith, 2000). Its related traits for example FL, FS, and FM are more important for the spinning industry. Previous investigations had shown that FL and FS have significant negative correlations with earliness in cotton. Thus, the early-maturity cotton varieties have much lower fiber quality than late-maturity ones. Sun et al. (2017) reported the association panel including early-, middle- and late- maturity cotton varieties have a big phenotypic variation of the FL (22.07~35.56 mm) and FS (22.69~36.80 cN.tex^−1^). In this study, FL of the panel ranged from 24.07 to 33.69 mm, with a mean of 28.09 mm; while the FS had a great variation ranging from 22.70 to 40.65 cN.tex^−1^. These findings indicate that FL of our association population of the early-maturity cotton has small distribution ranges compared with the previous results.

Although China is one of the largest nations producing and consuming cotton in the world, it is not an upland cotton domestication country (Zhang et al., 2013). The early cotton varieties were primarily developed by using introduced varieties (Zhang et al., 2013). King cultivar from America is the ancestor of the Chinese early-maturity upland cotton. Most of Chinese early-maturity cotton varieties of the early stage, such as “Jinmian1,” “Heishanmian1,” “Liaomian1,” “Zhongmiansuo10,” and “Xinluzao10,” were all derived from “Guannong1,” which had a breeding pedigree from the King cultivar. In this study, the association panel contained the above-mentioned core germplasms, and consisted of more than 80% of the Chinese early-maturity cotton varieties. Thus, it can represent the wide genetic diversity of Chinese early-maturity upland cotton. In the early stage, the Chinese early-maturity cotton varieties were developed by utilizing the core germplasms from NSEMR (“Jinmian1,” “Heishanmian1” and “Liaomian1). On the basis of the clustering of phylogenetic tree and PCA of the study, along with breeding history, the early-maturity cotton could be divided into three groups, designated pop I (the recent accessions from YRR), pop II (the recent accessions from NIR) and pop III (the NSEMR varieties and the early germplasms from YRR and NIR), respectively. These findings suggest that early-maturity accessions in YRR and NIR might derive from the NSEMR early varieties in Chinese upland cotton.

### Comparison of our GWAS results with QTL or QTNs detected in previous studies

In the recent 30 years, many QTL have been mapped, and some fiber-quality QTL hotspots have been discovered by a comparative meta-analysis (Said et al., 2015). It has been shown that chromosome D11 (c24) has the most prominent cluster carrying FL, FE and FS QTL hotspots between CIR026 and NAU2407b. A hotspot cluster A07 (c7) carrying FL and FS QTL between E1M7_80 and CG05a has also been found (Said et al., 2015). Another cluster carrying FE, FL and FM QTL hotspots on D01 (c14) between CIR246 and G1012 has been identified; and the region between E5M4_480 and pAR544 harbors a hotspot cluster carrying FS QTL on chromosome D03 (c16) (Said et al., 2015). Additionally, some stable QTL for FS on A07 (Chr.07) have been identified by QTL mapping (Tan et al., 2015; Fang X. et al., 2017). Similarly, a few associated SNP loci with fiber quality have been detected via GWAS in upland cotton (Table S4). Among the identified FL-associated SNPs, most of markers were located on chromosome A10 and D11, such as A10_65694094, A10_65696540, D11_24030081 and D11_24030087. Recent reports have shown a number of cluster_A07 SNPs for FS are distributed in genome region A07: 71.99–72.25 Mbp (Sun et al., 2017; Ma Z. et al., 2018). In addition, we also found the major genomic region (D11:24.03–24.10 Mbp) consisting of nine SNP loci associated with FL, which was previously detected (Su et al., 2016a).

In the current study, we characterized the significant QTNs (D11_21619830, A05_28352019 and D03_34920546) for fiber-quality related traits. These QTNs were detected using several new ML-GWAS methods in at least two environments. Compared with the mapped QTL of the previous studies, the QTN D11_21619830 was located in the region of QTL hotspot clusters for fiber quality. Compared with the associated loci of previous GWAS, these associated QTNs were excluded in the genomic regions of the previous reports. Therefore, these identified SNPs may be novel QTNs controlling fiber quality in our association population of early-maturity cotton.

### Superiority of the new multi-locus GWAS

Most of previous studies have focused on genetic bases of some complicated traits using general linear model (GLM) and mixed linear model (MLM) based on a single-locus GWAS (SL-GWAS) (Yu et al., 2006; Zhang et al., 2010). However, both of these models have certain shortcomings. A big false positive incidence is the uppermost disadvantage of GLM because polygenic kinship is not considered (Korte and Farlow, 2013). In MLM, the stringent *P* threshold (*P* = 0.05/*n, n* is the number of SNPs) leads to missing many significant QTNs, particularly small-effect QTNs (Wang et al., 2016). To make up for deficiencies of GLM and MLM, some multi-locus GWAS (ML-GWAS) methodologies have been developed, such as mrMLM (Wang et al., 2016), FASTmrMLM (Tamba and Zhang, 2018), FASTmrEMMA (Wen et al., 2017), ISIS EM-BLASSO (Tamba et al., 2017), pLARmEB (Zhang et al., 2017), and pKWmEB (Ren et al., 2018). Compared with the conventional SL-GWAS MLM methods, these ML-GWAS methods are more powerful and have the advantages of accuracy. Thus, we adopted the ML-GWAS methods in this study.

In addition, the significant threshold of these new ML-GWAS methods is set to a LOD score = 3, which is equal to –lg(*P*) = 3.70 (Wang et al., 2016). Although the standards are less stringent in the ML-GWAS methods than in the SL-MLM ones, their false positive rates are effectively reduced (Wang et al., 2016; Tamba et al., 2017; Wen et al., 2017; Zhang et al., 2017; Ren et al., 2018; Tamba and Zhang, 2018). Thus, the ML-GWAS approaches are considered more effective, practical and alternative. In this study, 70 QTNs significantly associated with five fiber-quality related traits were simultaneously identified in three or more ML-GWAS methods (Table 3). Further investigation showed that three stably expressed QTNs were commonly detected in multiple environments (Table 3). However, no significantly associated QTN was found when using the Tassel 5.0 in MLM [PCs + K, –lg(*P*) = –lg(0.05/72792) = 6.16]. These data suggest that the ML-GWAS methods are more powerful and robust when applying to detect the small-effect QTNs for fiber-quality related traits of upland cotton.

## Conclusion

In this study, a total of 70 significant QTNs were simultaneously detected to be associated with five objective traits by three or more methods. Among these QTNs, D11_21619830, A05_28352019 and D03_34920546, significantly associated with FL, FS, and FM, respectively, were simultaneously presented across at least two environments. Furthermore, favorable allelic variations of the three QTNs and 96 genes contained in the three target genomic range were mined. Among these, six genes highly expressed in the fibers might be candidate genes identified by RNA-Seq method. In summary, many favorable QTN alleles and six candidate genes were identified to modulate fiber development in early-maturity upland cotton. This will lay a solid basis for breeding earliness and excellent fiber-quality cotton varieties in the future.

## Availability of supporting data

The sequence read data from the SLAF-seq analysis for the 160 sequenced upland cotton lines are available in the Sequence Read Archive (http://www.ncbi.nlm.nih.gov/bioproject/PRJNA314284/) (SRP071133 under the accession number PRJNA314284).

## Author contributions

JS and CW designed and supervised the research. JS and ML performed multi-locus GWAS. JS and QM conducted the field trials to evaluate the five fiber-quality related traits. JS, CW, and FH wrote and revised the manuscript. All of the authors read and approved the manuscript.

### Conflict of interest statement

The authors declare that the research was conducted in the absence of any commercial or financial relationships that could be construed as a potential conflict of interest.
