# Supplementary material for: Multi-Locus Genome-Wide Association Studies of Fiber-Quality Related Traits in Chinese Early-Maturity Upland Cotton
Source: Front Plant Sci. 2018 Aug 16;9:1169. doi: 10.3389/fpls.2018.01169 (PMC6107031; doi:10.3389/fpls.2018.01169)
Supplement: Supplementary file 1 [file Table_1.DOCX]

Supplementary Table S1. Information of the 160 early-maturity upland cotton accessions

YYR, Yellow River Region;

NIR, Northwest Inland Region;

NSEMR, Northern Specific Early-Maturity Region.

| Serial  number | Variety  names | Geographic  origin | Serial  number | Variety  names | Geographic  origin | Serial  number | Variety  names | Geographic  origin | Serial  number | Variety  names | Geographic  origin |
| --- | --- | --- | --- | --- | --- | --- | --- | --- | --- | --- | --- |
| 1 | zhongmianzao0191 | YYR | 41 | Jinmian23 | YYR | 81 | zhong716 | YYR | 121 | xinluzao27 | NIR |
| 2 | zhongmianzao0418 | YYR | 42 | Jinmian5 | YYR | 82 | zhong751213 | YYR | 122 | xinluzao28 | NIR |
| 3 | zhongmianzao0618 | YYR | 43 | liaomian10 | NSEMR | 83 | zhongmiansuo10 | YYR | 123 | xinluzao29 | NIR |
| 4 | zhongmianzao0712 | YYR | 44 | liaomian17 | NSEMR | 84 | zhongmiansuo14 | YYR | 124 | xinluzao30 | NIR |
| 5 | zhongmianzao0811 | YYR | 45 | liaomian5 | NSEMR | 85 | zhongmiansuo16 | YYR | 125 | xinluzao32 | NIR |
| 6 | zhongmianzao0822 | YYR | 46 | liaomian6 | NSEMR | 86 | zhongmiansuo20 | YYR | 126 | xinluzao33 | NIR |
| 7 | zhongmianzao1832 | YYR | 47 | liaomian7 | NSEMR | 87 | zhongmiansuo24 | YYR | 127 | xinluzao34 | NIR |
| 8 | zhongmianzao1239 | YYR | 48 | liaomian9 | NSEMR | 88 | zhongmiansuo27 | YYR | 128 | xinluzao35 | NIR |
| 9 | PB12-1-10 | YYR | 49 | liaomianduanjie | NSEMR | 89 | zhongmiansuo30 | YYR | 129 | xinluzao37 | NIR |
| 10 | PB12-1-7 | YYR | 50 | lu154 | YYR | 90 | zhongmiansuo36 | YYR | 130 | xinluzao38 | NIR |
| 11 | PB12-1-8 | YYR | 51 | lu890 | YYR | 91 | han256 | YYR | 131 | xinluzao39 | NIR |
| 12 | zhongmianzao1476 | YYR | 52 | lumian2153 | YYR | 92 | zhongmiansuo37 | YYR | 132 | xinluzao40 | NIR |
| 13 | zhongmianzao1222 | YYR | 53 | nongken5 | NIR | 93 | zhongmiansuo42 | YYR | 133 | xinluzao41 | NIR |
| 14 | zhongmianzao2201 | YYR | 54 | shan70 | YYR | 94 | zhongmiansuo50 | YYR | 134 | xinluzao46 | NIR |
| 15 | SQ152201 | YYR | 55 | shizao1 | YYR | 95 | zhongmiansuo58 | YYR | 135 | xinluzao47 | NIR |
| 16 | zhongmianzao2214 | YYR | 56 | shizao2 | YYR | 96 | zhongmiansuo64 | YYR | 136 | xinluzao48 | NIR |
| 17 | SQ152224 | YYR | 57 | shizao3 | YYR | 97 | zhongmiansuo74 | YYR | 137 | xinluzao49 | NIR |
| 18 | 2011SS | YYR | 58 | xia13-7 | YYR | 98 | liaomian23 | NSEMR | 138 | xinluzao50 | NIR |
| 19 | QS2012-3 | YYR | 59 | xia25 | YYR | 99 | liaomian27 | NSEMR | 139 | xinluzao51 | NIR |
| 20 | QS2012-4 | YYR | 60 | xiazao1 | YYR | 100 | liaomian28 | NSEMR | 140 | xinluzao60 | NIR |
| 21 | 29-41 | YYR | 61 | xiazao2 | YYR | 101 | xinluzao2 | NIR | 141 | huiyuan717 | NIR |
| 22 | 29-42 | YYR | 62 | xiazao3 | YYR | 102 | xinluzao10 | NIR | 142 | yunzao219 | YYR |
| 23 | 6426 | YYR | 63 | xinluzao11 | NIR | 103 | xinluzao12 | NIR | 143 | yunzao33-356 | YYR |
| 24 | K640 | YYR | 64 | xinluza | NIR | 104 | xinluzao13 | NIR | 144 | jinmian2 | NSEMR |
| 25 | N82 | YYR | 65 | xinluzao3 | NIR | 105 | xinluzao15 | NIR | 145 | chaoyangmian2 | NSEMR |
| 26 | baimian17 | YYR | 66 | xinluzao42 | NIR | 106 | xinluzao16 | NIR | 146 | dunhuang77-116 | NIR |
| 27 | chaoyangmian1 | NSEMR | 67 | xinluzao45 | NIR | 107 | xinluzao17 | NIR | 147 | ganmian4 | NIR |
| 28 | Deltapine20 | NSEMR | 68 | xinluzao4 | NIR | 108 | xinluzao18 | NIR | 148 | guannongzaoC-50 | NSEMR |
| 29 | Delfos97-047 | NSEMR | 69 | xinluzao6 | NIR | 109 | xinluzao19 | NIR | 149 | guannongchangzao14 | NSEMR |
| 30 | guannong1 | NSEMR | 70 | xinluzao8 | NIR | 110 | xinluzao20 | NIR | 150 | yanzao1 | YYR |
| 31 | han2490 | YYR | 71 | xinluzao9 | NIR | 111 | xinluzao21 | NIR | 151 | yanzao2 | YYR |
| 32 | han656 | YYR | 72 | xinxiang368 | YYR | 112 | xinluzao22 | NIR | 152 | yiaojinmian6 | NSEMR |
| 33 | han559 | YYR | 73 | yu1335 | YYR | 113 | xinluzao23 | NIR | 153 | jinken69-2 | NIR |
| 34 | han667 | YYR | 74 | yumian12 | YYR | 114 | xinluzao24 | NIR | 154 | jinken148-39 | NIR |
| 35 | han686 | YYR | 75 | yuzao8E13 | YYR | 115 | yumian5 | YYR | 155 | 611bo | NIR |
| 36 | han9609 | YYR | 76 | yuzao910 | YYR | 116 | yumian18 | YYR | 156 | dunmian1 | NIR |
| 37 | heishanmian1 | NSEMR | 77 | yunzaoN177 | YYR | 117 | bo425 | NIR | 157 | dunmian2 | NIR |
| 38 | Jingmian3 | NSEMR | 78 | yunzaoN95 | YYR | 118 | kenN27-3 | NIR | 158 | ganmian2 | NIR |
| 39 | Jinmian10 | YYR | 79 | zhong416 | YYR | 119 | xinluzao25 | NIR | 159 | keke1543 | NIR |
| 40 | Jinmian21 | YYR | 80 | zhong425-5 | YYR | 120 | xinluzao26 | NIR | 160 | xinluzao53 | NIR |
