# Supplementary material for: Multi-Locus Genome-Wide Association Studies of Fiber-Quality Related Traits in Chinese Early-Maturity Upland Cotton
Source: Front Plant Sci. 2018 Aug 16;9:1169. doi: 10.3389/fpls.2018.01169 (PMC6107031; doi:10.3389/fpls.2018.01169)
Supplement: Supplementary file 2 [file Table_2.DOCX]

| Table S2 Analysis of variance (ANOVA) of the five fiber-quality related traits of 160 lines  in four environments. |
| --- |
| \| Traits \| Influencing factors \| SS \| df \| MS \| F \| Sig. \| \| --- \| --- \| --- \| --- \| --- \| --- \| --- \| \| FL \| Genotypes (G) \| 2982.218 \| 159 \| 18.756 \| 16.086 \| 0.000 \| \|  \| Environment (E) \| 1002.758 \| 3 \| 334.253 \| 286.660 \| 0.000 \| \|  \| G×E interactions \| 964.491 \| 477 \| 2.022 \| 1.734 \| 0.000 \| \| FS \| Genotypes (G) \| 10251.807 \| 159 \| 64.477 \| 28.119 \| 0.000 \| \|  \| Environment (E) \| 5250.145 \| 3 \| 1750.048 \| 763.219 \| 0.000 \| \|  \| G×E interactions \| 2446.729 \| 477 \| 5.129 \| 2.237 \| 0.000 \| \| FM \| Genotypes (G) \| 258.467 \| 159 \| 1.626 \| 18.932 \| 0.000 \| \|  \| Environment (E) \| 46.156 \| 3 \| 15.385 \| 179.179 \| 0.000 \| \|  \| G×E interactions \| 98.872 \| 477 \| 0.207 \| 2.414 \| 0.000 \| \| FU \| Genotypes (G) \| 2340.239 \| 159 \| 14.718 \| 2.860 \| 0.000 \| \|  \| Environment (E) \| 327.931 \| 3 \| 109.310 \| 21.243 \| 0.000 \| \|  \| G×E interactions \| 2825.587 \| 477 \| 5.924 \| 1.151 \| 0.030 \| \| FE \| Genotypes (G) \| 8.414 \| 159 \| 0.053 \| 1.838 \| 0.000 \| \|  \| Environment (E) \| 73.990 \| 3 \| 24.663 \| 856.880 \| 0.000 \| \|  \| G×E interactions \| 24.863 \| 477 \| 0.052 \| 1.811 \| 0.000 \| |
