# Supplementary material for: Multi-Locus Genome-Wide Association Studies of Fiber-Quality Related Traits in Chinese Early-Maturity Upland Cotton
Source: Front Plant Sci. 2018 Aug 16;9:1169. doi: 10.3389/fpls.2018.01169 (PMC6107031; doi:10.3389/fpls.2018.01169)
Supplement: Supplementary file 3 [file Table_3.DOC]

| Table S3 Information of 96 genes contained in the three target regions |
| --- |
| | **Gene ID** | **Start** | **End** | **Gene Name** | **Description** | | --- | --- | --- | --- | --- | | Gh_A05G2319 | 27,756,669 | 27,759,159 | UDP-GALT2 | UDP-galactose transporter 2 | | Gh_A05G2320 | 27,763,189 | 27,765,178 | RPS18A | 40S ribosomal protein S18 | | Gh_A05G2321 | 27,807,410 | 27,817,637 | SOL1 | Carboxypeptidase SOL1 | | Gh_A05G2322 | 27,836,442 | 27,844,832 | CK1 | Probable choline kinase 1 | | Gh_A05G2323 | 27,853,463 | 27,859,900 | IP5P3 | Type IV inositol polyphosphate 5-phosphatase 3 | | Gh_A05G2324 | 27,887,458 | 27,891,958 | At1g34110 | Probable LRR receptor-like serine/threonine-protein kinase At1g34110 | | Gh_A05G2325 | 27,895,324 | 27,895,584 | NA | Non-specific lipid-transfer protein 2 | | Gh_A05G2326 | 27,898,063 | 27,902,166 | NA | Ras-related protein Rab7 | | Gh_A05G2327 | 27,904,650 | 27,907,646 | At4g09670 | Uncharacterized oxidoreductase At4g09670 | | Gh_A05G2328 | 27,922,427 | 27,923,758 | LAC14 | Laccase-14 | | Gh_A05G2329 | 28,004,172 | 28,004,746 | NA | NA | | Gh_A05G2330 | 28,017,577 | 28,021,852 | YLR455W | PWWP domain-containing protein YLR455W | | Gh_A05G2331 | 28,068,826 | 28,069,477 | EPF2 | Protein EPIDERMAL PATTERNING FACTOR 2 | | Gh_A05G2332 | 28,264,059 | 28,267,109 | IKU2 | Receptor-like protein kinase HAIKU2 | | Gh_A05G2333 | 28,271,103 | 28,273,816 | CCR4 | Serine/threonine-protein kinase-like protein CCR4 | | Gh_A05G2334 | 28,279,032 | 28,282,673 | AGL11 | Agamous-like MADS-box protein AGL11 | | Gh_A05G2335 | 28,311,283 | 28,313,447 | PATL3 | Patellin-3 | | Gh_A05G2336 | 28,356,753 | 28,359,007 | PATL5 | Patellin-5 | | Gh_A05G2337 | 28,400,340 | 28,402,319 | NPF5.10 | Protein NRT1/ PTR FAMILY 5.10 | | Gh_A05G2338 | 28,473,145 | 28,475,873 | QKY | Protein QUIRKY | | Gh_A05G2339 | 28,476,738 | 28,485,707 | cdkal1 | Threonylcarbamoyladenosine tRNA methylthiotransferase | | Gh_A05G2340 | 28,486,992 | 28,487,615 | CG12206 | Glutaredoxin domain-containing cysteine-rich protein CG12206 | | Gh_A05G2341 | 28,510,191 | 28,512,581 | Gtf3a | Transcription factor IIIA | | Gh_A05G2342 | 28,567,167 | 28,568,192 | MYB6 | Transcription repressor MYB6 | | Gh_A05G2343 | 28,648,101 | 28,649,369 | TCP23 | Transcription factor TCP23 | | Gh_A05G2344 | 28,659,193 | 28,659,972 | YLS9 | Protein YLS9 | | Gh_A05G2345 | 28,662,292 | 28,665,925 | CINV2 | Alkaline/neutral invertase CINV2 | | Gh_A05G2346 | 28,687,889 | 28,688,137 | ARG7 | Indole-3-acetic acid-induced protein ARG7 | | Gh_A05G2347 | 28,700,915 | 28,701,560 | PER43 | Peroxidase 43 | | Gh_D03G1011 | 34,550,221 | 34,552,065 | AKR4C9 | Aldo-keto reductase family 4 member C9 | | Gh_D03G1012 | 34,597,931 | 34,600,275 | CNR6 | Cell number regulator 6 | | Gh_D03G1013 | 34,602,248 | 34,609,360 | SMC2-1 | Structural maintenance of chromosomes protein 2-1 | | Gh_D03G1014 | 34,646,654 | 34,648,309 | At3g47420 | Putative glycerol-3-phosphate transporter 1 | | Gh_D03G1015 | 34,650,899 | 34,651,264 | At3g47420 | Putative glycerol-3-phosphate transporter 1 | | Gh_D03G1016 | 34,653,943 | 34,654,113 | NA | NA | | Gh_D03G1017 | 34,661,789 | 34,664,900 | WAK2 | Wall-associated receptor kinase 2 | | Gh_D03G1018 | 34,682,919 | 34,685,125 | BAG7 | BAG family molecular chaperone regulator 7 | | Gh_D03G1019 | 34,714,659 | 34,716,838 | TPPA | Trehalose-phosphate phosphatase A | | Gh_D03G1020 | 34,733,946 | 34,737,262 | INV*DC4 | Beta-fructofuranosidase, soluble isoenzyme I | | Gh_D03G1021 | 34,752,815 | 34,754,411 | NAC007 | NAC domain-containing protein 7 | | Gh_D03G1022 | 34,773,019 | 34,776,739 | At5g62370 | Pentatricopeptide repeat-containing protein At5g62370 | | Gh_D03G1023 | 34,777,576 | 34,777,917 | PECS-2.1 | Pectinesterase 2 | | Gh_D03G1024 | 34,778,858 | 34,779,202 | PECS-2.1 | Pectinesterase 2 | | Gh_D03G1025 | 34,792,987 | 34,794,652 | PME33 | Probable pectinesterase/pectinesterase inhibitor 33 | | Gh_D03G1026 | 34,833,862 | 34,834,467 | NA | 21 kDa protein | | Gh_D03G1027 | 34,854,635 | 34,854,966 | NA | NA | | Gh_D03G1028 | 34,882,139 | 34,882,550 | NA | UTP--glucose-1-phosphate uridylyltransferase | | Gh_D03G1029 | 34,897,415 | 34,898,062 | NA | 21 kDa protein | | Gh_D03G1030 | 34,931,553 | 34,932,562 | PHO1-H1 | Phosphate transporter PHO1 homolog 1 | | Gh_D03G1031 | 34,964,505 | 34,965,095 | NA | 21 kDa protein | | Gh_D03G1032 | 35,019,420 | 35,030,473 | SP2L | Microtubule-associated protein SPIRAL2-like | | Gh_D03G1033 | 35,067,727 | 35,068,821 | CYP26-2 | Peptidyl-prolyl cis-trans isomerase CYP26-2, chloroplastic | | Gh_D03G1034 | 35,074,387 | 35,075,029 | NAD-ME2 | NAD-dependent malic enzyme 2, mitochondrial | | Gh_D03G1035 | 35,136,531 | 35,138,037 | TUFB1 | Elongation factor Tu, chloroplastic | | Gh_D03G1036 | 35,141,519 | 35,147,970 | At4g24710 | Pachytene checkpoint protein 2 homolog | | Gh_D03G1037 | 35,153,832 | 35,154,125 | NA | NA | | Gh_D03G1038 | 35,226,830 | 35,227,312 | NA | NA | | Gh_D03G1039 | 35,228,371 | 35,228,715 | NA | NA | | Gh_D03G1040 | 35,229,811 | 35,233,143 | HT1 | Serine/threonine-protein kinase HT1 | | Gh_D03G1041 | 35,265,771 | 35,266,176 | NA | NA | | Gh_D03G1042 | 35,274,434 | 35,277,944 | NBR1 | Protein NBR1 homolog | | Gh_D11G1848 | 21,124,848 | 21,127,654 | NA | Putative glucose-6-phosphate 1-epimerase | | Gh_D11G1849 | 21,141,907 | 21,144,894 | PUB5 | U-box domain-containing protein 5 | | Gh_D11G1850 | 21,209,864 | 21,214,065 | atad3-a | ATPase family AAA domain-containing protein 3-A | | Gh_D11G1851 | 21,238,006 | 21,238,980 | ZHD6 | Zinc-finger homeodomain protein 6 | | Gh_D11G1852 | 21,282,562 | 21,287,461 | At1g75220 | Sugar transporter ERD6-like 6 | | Gh_D11G1853 | 21,313,622 | 21,315,306 | ephx3 | Epoxide hydrolase 3 | | Gh_D11G1854 | 21,365,018 | 21,365,853 | GATA18 | GATA transcription factor 18 | | Gh_D11G1855 | 21,366,753 | 21,367,280 | NA | NA | | Gh_D11G1856 | 21,377,694 | 21,384,389 | nol6 | Nucleolar protein 6 | | Gh_D11G1857 | 21,385,456 | 21,386,821 | ARL2 | ADP-ribosylation factor-like protein 2 | | Gh_D11G1858 | 21,396,626 | 21,397,420 | METTL21C | Protein-lysine methyltransferase METTL21C | | Gh_D11G1859 | 21,397,976 | 21,403,166 | Vps39 | Vam6/Vps39-like protein | | Gh_D11G1860 | 21,414,041 | 21,414,211 | NA | NA | | Gh_D11G1861 | 21,421,379 | 21,421,798 | NA | NA | | Gh_D11G1862 | 21,463,124 | 21,466,296 | CYP90B1 | Cytochrome P450 90B1 | | Gh_D11G1863 | 21,502,521 | 21,505,813 | NA | NA | | Gh_D11G1864 | 21,601,361 | 21,602,581 | At5g47070 | Probable receptor-like protein kinase At5g47070 | | Gh_D11G1865 | 21,606,545 | 21,609,367 | PERK4 | Proline-rich receptor-like protein kinase PERK4 | | Gh_D11G1866 | 21,610,629 | 21,616,705 | At5g49610 | F-box protein At5g49610 | | Gh_D11G1867 | 21,621,038 | 21,621,529 | NA | NA | | Gh_D11G1868 | 21,657,570 | 21,660,272 | At3g50690 | Acidic leucine-rich nuclear phosphoprotein 32-related protein | | Gh_D11G1869 | 21,661,439 | 21,664,217 | gtf2b | Transcription initiation factor IIB | | Gh_D11G1870 | 21,767,311 | 21,769,606 | NA | NA | | Gh_D11G1871 | 21,779,536 | 21,787,189 | SEC11A | Signal peptidase complex catalytic subunit SEC11A | | Gh_D11G1872 | 21,817,593 | 21,822,215 | POLR3E | DNA-directed RNA polymerase III subunit RPC5 | | Gh_D11G1873 | 21,828,286 | 21,830,487 | IDD1 | Protein indeterminate-domain 1 | | Gh_D11G1874 | 21,832,473 | 21,834,814 | LAC6 | Laccase-6 | | Gh_D11G1875 | 21,837,664 | 21,840,364 | At5g66720 | Probable protein phosphatase 2C 80 | | Gh_D11G1876 | 21,840,716 | 21,846,315 | GBF1 | G-box-binding factor 1 | | Gh_D11G1877 | 21,848,462 | 21,852,293 | HT1 | Serine/threonine-protein kinase HT1 | | Gh_D11G1878 | 21,857,868 | 21,858,656 | At5g40230 | WAT1-related protein At5g40230 | | Gh_D11G1879 | 22,013,016 | 22,014,350 | ATHB-40 | Homeobox-leucine zipper protein ATHB-40 | | Gh_D11G1880 | 22,033,011 | 22,034,760 | At4g36750 | Probable NAD(P)H dehydrogenase (quinone) FQR1-like 2 | | Gh_D11G1881 | 22,071,828 | 22,074,080 | NA | NA | | Gh_D11G1882 | 22,074,853 | 22,076,011 | RCE1 | NEDD8-conjugating enzyme Ubc12 | |
