# Supplementary material for: Multi-Locus Genome-Wide Association Studies of Fiber-Quality Related Traits in Chinese Early-Maturity Upland Cotton
Source: Front Plant Sci. 2018 Aug 16;9:1169. doi: 10.3389/fpls.2018.01169 (PMC6107031; doi:10.3389/fpls.2018.01169)
Supplement: Supplementary file 4 [file Table_4.DOCX]

| Table S4 Information of the significant SNPs associated with FL and FS  in previous GWAS in upland cotton |
| --- |
| \| Traits \| Sun et al. 2017 \| \| Su et al. 2016 \| \| Ma et al. 2016 \| \| \| --- \| --- \| --- \| --- \| --- \| --- \| --- \| \|  \| Chro. \| SNP loci \| Chro. \| SNP loci \| Chro. \| SNP loci \| \| FL \| A07 \| 72067994 \| D11 \| 24034609 \| A10 \| 65694094 \| \|  \| A07 \| 72193182 \| D11 \| 24034607 \| A10 \| 65696540 \| \|  \| A07 \| 72198802 \| D11 \| 24056372 \| D11 \| 24030087 \| \|  \| A07 \| 72200974 \| D11 \| 24056611 \|  \|  \| \|  \| A07 \| 72204773 \| D11 \| 24067326 \|  \|  \| \|  \| A07 \| 72213592 \| D11 \| 24073931 \|  \|  \| \|  \| A07 \| 72249786 \| D11 \| 24074137 \|  \|  \| \|  \| D11 \| 23906867 \| D11 \| 24102240 \|  \|  \| \|  \| D11 \| 23959318 \|  \|  \|  \|  \| \|  \| D11 \| 24008823 \|  \|  \|  \|  \| \| FS \| A07 \| 71993462 \|  \|  \| A07 \| 72204322 \| \|  \| A07 \| 72008085 \|  \|  \| A07 \| 72204443 \| \|  \| A07 \| 72067994 \|  \|  \|  \|  \| \|  \| A07 \| 72193182 \|  \|  \|  \|  \| \|  \| A07 \| 72198802 \|  \|  \|  \|  \| \|  \| A07 \| 72200974 \|  \|  \|  \|  \| \|  \| A07 \| 72204773 \|  \|  \|  \|  \| \|  \| A07 \| 72213592 \|  \|  \|  \|  \| \|  \| A07 \| 72249786 \|  \|  \|  \|  \| \|  \| D11 \| 24030081 \|  \|  \|  \|  \| |
| Sun, Z. W., Wang, X. F., Liu, Z. W., Gu, Q. S., Zhang, Y., Li, Z. K., et al. (2017). Genome-wide association study discovered genetic variation and candidate genes of fibre quality traits in Gossypium hirsutum L. Plant Biotechnol J. 1, 1-15. doi: 10.1111/pbi.12693  Su, J. J., Li, L. B., Pang, C. Y., Wei, H. L., Wang, C. X., Song, M. Z., et al. (2016a). Two genomic regions associated with fiber quality traits in chinese upland cotton under apparent breeding selection. Sci. Rep. 6: 38496. doi: 10.1038/srep 38496  Ma, Z. Y., He, S. P., Wang, X. F., Sun, J. L., Zhang, Y., Zhang, G. Y. et al. (2018b). Resequencing a core collection of upland cotton identifies genomic variation and loci influencing fiber quality and yield. Nat. Genet. 50, 803-813. doi: 10.1038/s41588-018-0119-7 |
